# A novel *C. elegans* respirometry assay using low-cost optical oxygen sensors

**DOI:** 10.1093/biomethods/bpaf072

**Published:** 2025-09-30

**Authors:** Nathan Dennis, Campbell W Gourlay, Marina Ezcurra

**Affiliations:** School of Natural Sciences, University of Kent, Canterbury, CT2 7NZ, United Kingdom; School of Natural Sciences, University of Kent, Canterbury, CT2 7NZ, United Kingdom; School of Natural Sciences, University of Kent, Canterbury, CT2 7NZ, United Kingdom

**Keywords:** *C. elegans*, respirometry, mitochondrial function

## Abstract

Measurement of the oxygen consumption rate, or respirometry, is a powerful and comprehensive method for assessing mitochondrial function both *in vitro* and *in vivo*. Respirometry at the whole-organism level has been repeatedly performed in the model organism *Caenorhabditis elegans*, typically using high-throughput microplate-based systems over traditional Clark-type respirometers. However, these systems are highly specialized, costly to purchase and operate, and inaccessible to many researchers. Here, we develop a respirometry assay using low-cost commercially available optical oxygen sensors (PreSens OxoPlates^®^) and fluorescence plate readers (the BMG FLUOstar), as an alternative to more costly standard respirometry systems. This assay uses standard BMG FLUOstar protocols and a set of custom scripts to perform repeated measurements of the *C. elegans* oxygen consumption rate, with the optional use of respiratory inhibitors or other interventions. We validate this assay by demonstrating the linearity of basal oxygen consumption rates in samples with variable numbers of animals, and by examining the impact of respiratory inhibitors with previously demonstrated efficacy in *C. elegans*: carbonyl cyanide 4-(trifluoromethoxy) phenylhydrazone (a mitochondrial uncoupler) and sodium azide (a Complex IV inhibitor). Using this assay, we demonstrate that the sequential use of FCCP and sodium azide leads to an increase in the sodium azide-treated (non-mitochondrial) oxygen consumption rate, indicating that the sequential use of respiratory inhibitors, as standard in intact cell respirometry, may produce erroneous estimates of non-mitochondrial respiration in *C. elegans* and thus should be avoided.

## Introduction

The mitochondria are central to cellular health, performing critical functions in metabolism, ion homeostasis, cell signalling, and ATP synthesis, among many other processes (reviewed in references [1, 2]). Consequently, mitochondrial dysfunction is associated with a diverse spectrum of human diseases, most notably genetic mitochondrial diseases [3], but also common and increasingly prevalent conditions such as Alzheimer’s disease, obesity, and type 2 diabetes [4–6].

Mitochondrial health can be examined via numerous methods, including the microscopic evaluation of mitochondrial morphology [7], estimation of the mitochondrial membrane potential [8], bioluminescence- or fluorescence-based ATP assays [9–11], and PCR-based assays of mtDNA copy number [12]. However, while each of these methods provides useful metrics of mitochondrial function, they fail to provide a complete picture of mitochondrial health.

**Figure 1:**
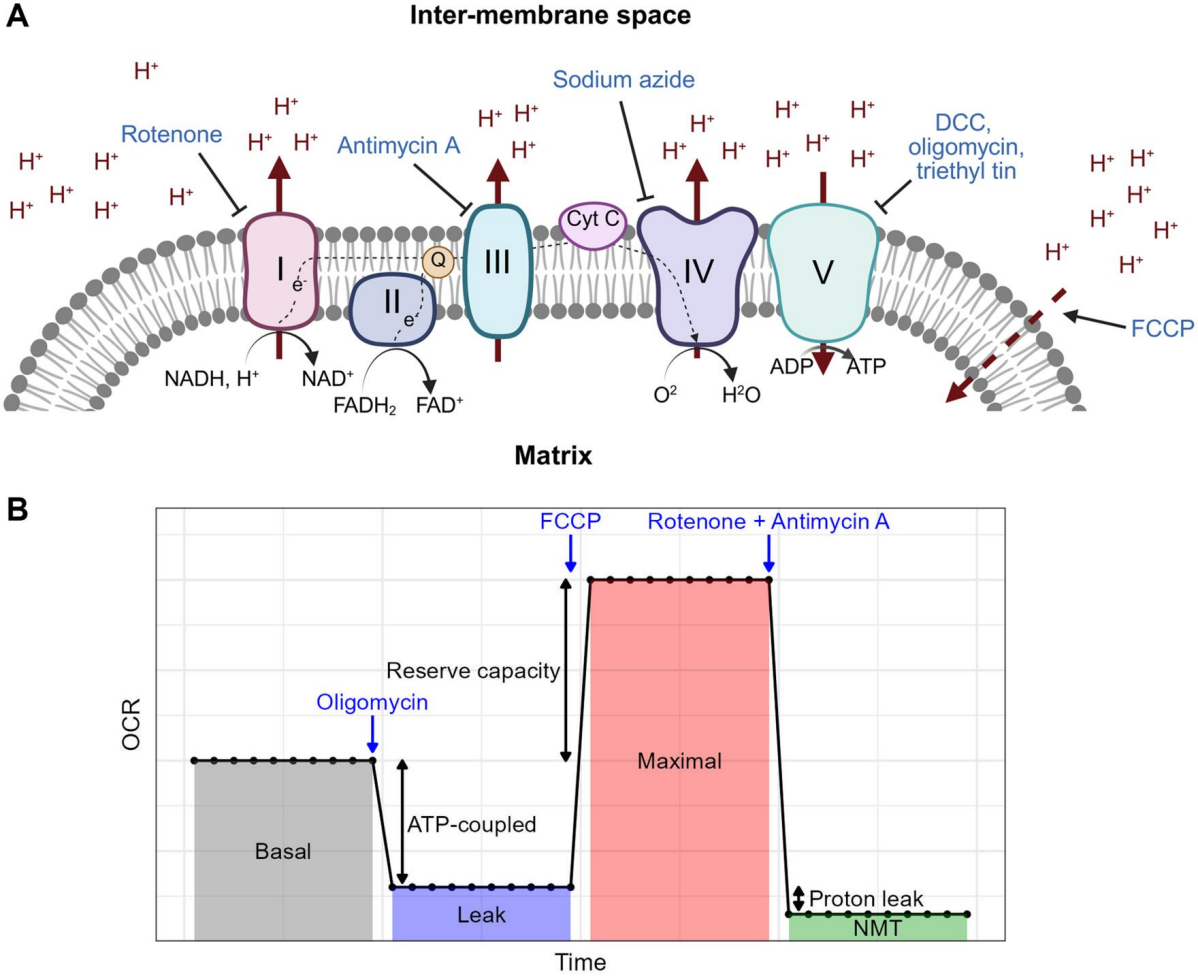
Overview of the mitochondrial electron transport chain and respiratory parameters. (A) Diagram of the mitochondrial electron transport chain with examples of respiratory inhibitors. I, Complex I; II, Complex II; III, Complex III; IV, Complex IV; V, Complex V (ATP synthase); DCC, dicyclohexylcarbodiimide; FCCP, carbonyl cyanide 4-(trifluoromethoxy) phenylhydrazone. Created in BioRender. Dennis, N. (2025) https://BioRender.com/8n4787c. (B) Overview of a typical respiratory profile. In a standard respirometry experiment with intact cells or whole organisms, initial OCR measurements are taken in an untreated state to assess the rate of basal respiration, followed by the addition of an ATP synthase inhibitor to measure the ATP synthase-inhibited (leak-state) OCR, an uncoupler to assess the maximal OCR, and a combination of a Complex I and Complex III inhibitor (typically rotenone and antimycin A) to assess the non-mitochondrial (NMT) OCR. Additional respiratory parameters can be calculated from these measurements, including the rate of ATP synthase-coupled respiration (the basal minus leak-state OCR), the reserve capacity (the maximal minus basal OCR), and the rate of proton leak across the inner mitochondrial membrane (the leak-state minus NMT OCR).

In contrast, measurement of the oxygen consumption rate (OCR), or respirometry, is the most direct and comprehensive method of examining cellular metabolism and mitochondrial function [13]. As the vast majority of oxygen consumption occurs via the mitochondrial electron transport chain (ETC; Fig. 1A), respirometry employing specific mitochondrial substrates or ETC inhibitors can provide informative readouts of mitochondrial function, such as the rates of ATP synthase-linked respiration, maximal respiration, and non-mitochondrial respiration [14] (Fig. 1B). OCRs can be measured in isolated mitochondria, intact or permeabilized cells, and 3D models (tissues, organoids or whole organisms), providing scope for the assessment of mitochondrial function in multiple contexts [13].

While respirometry in isolated mitochondria simplifies the interpretation of OCR data, experiments in whole organisms are more physiologically relevant, as they preserve the integrity of the mitochondrial network [15], their interactions with other cellular components, and the influence of intercellular signalling processes [16, 17]. This approach to respirometry has been repeatedly performed in the nematode *Caenorhabditis elegans* [18–28], elucidating the impact of genetic defects in electron transport chain subunits, fission-fusion dynamics and mitophagy [18], sex- and development-specific respiratory profiles [26, 28], and the utility of the OCR as a sub-lethal endpoint for toxicity screening [27].

Respirometry is generally performed using one of two types of devices: large, chamber-based Clark-type electrode systems, such as the Oroboros Oxygraph-2k; and small, specialized microplate-based systems using oxygen-sensitive phosphors, such as Agilent Seahorse extracellular flux (XF) analysers [13]. Respirometry studies in *C. elegans* have been performed using both Clark-type and microplate-based respirometers [18, 20, 24, 25], but the large requirement for biological material and low throughput associated with Clark-type respirometry [13] has led to a clear preference for the latter, as reflected in numerous *C. elegans* protocols outlining the use of the 8-well, 24-well and 96-well Seahorse XF analysers [19–23]. While the low requirement for biological material and high throughput of microplate-based systems make them ideally suited for work with *C. elegans*, these systems are associated with high upfront and ongoing costs [13, 29]. For many researchers, these costs are likely prohibitive, rendering microplate-based respirometry inaccessible.

**Figure 2:**
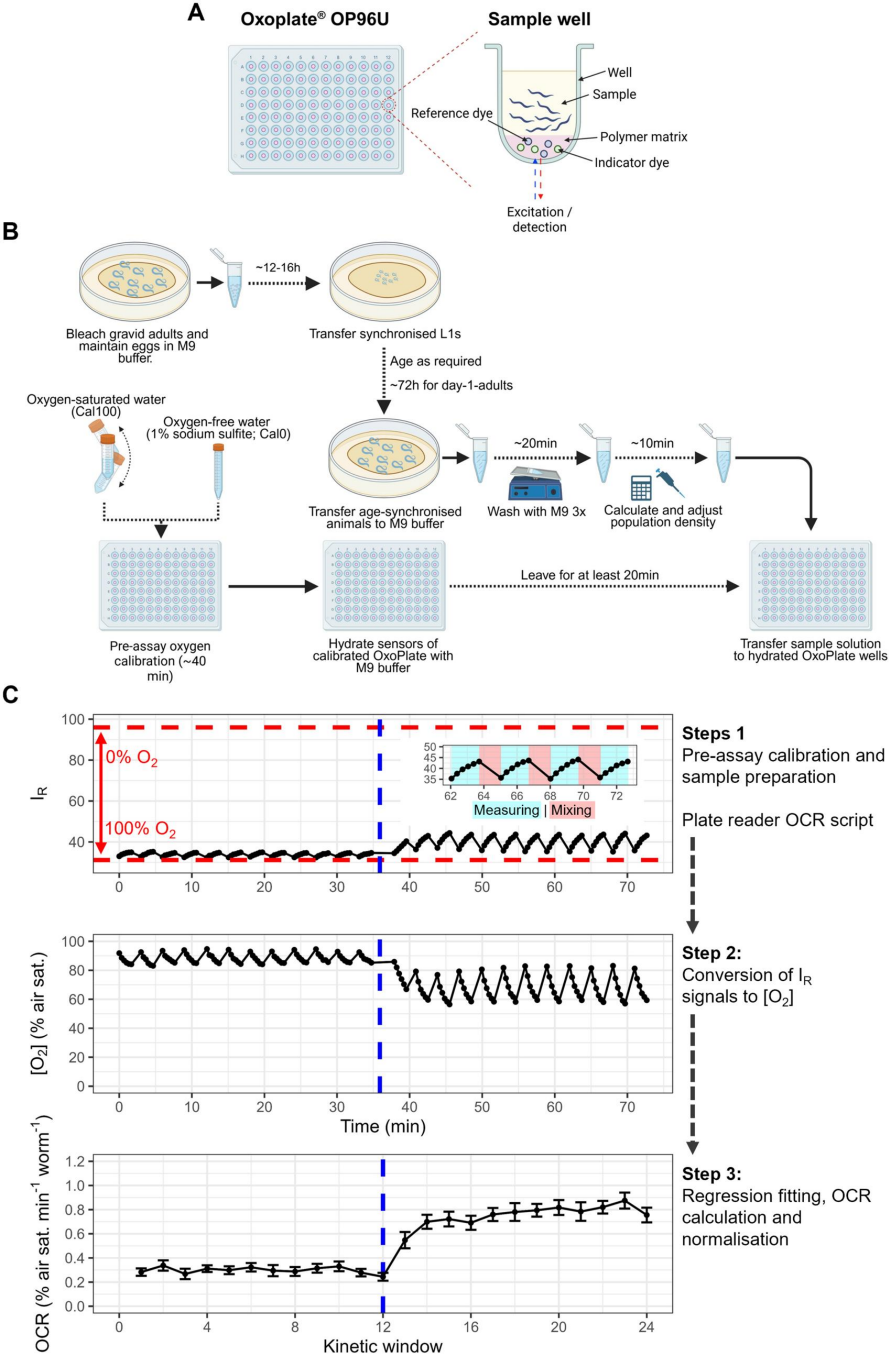
Graphical overview of the assay. (A) Diagram of an OxoPlate OP96U and sample well. (B) Graphical overview of *C. elegans* sample preparation and OxoPlate calibration. Panels A and B were created in BioRender. Dennis, N. (2025) https://BioRender.com/r5atevs. (C) Stepwise summary of a typical experiment. Step 1, Pre-assay sensor calibration (dashed red lines) and *C. elegans* sample preparation (as depicted in B). Step 2, A kinetic windows measurement protocol is looped by an oxygen consumption rate (OCR) script, measuring internally referenced (I_R_) sensor readouts every 20 s for 100 s (“measuring” in inset), interspersed with 1 min periods of 700 RPM linear shaking to re-oxygenate the sample wells (“mixing” in inset), generating repeated periods of oxygen consumption which are subsequently used to calculate the OCR. Step 3, I_R_ data are converted into oxygen concentrations using calibration data from step 1. Step 4, a data analysis script fits linear regressions to each period of oxygen consumption (kinetic window), calculates the OCR by extracting the slope of the regression, and normalizes the data using a user-defined parameter (here the number of animals per well). The data presented here are mean I_R_, [O_2_] and OCR (± SE) traces from an experiment using an optimal concentration of the uncoupler carbonyl cyanide 4-(trifluoromethoxy) phenylhydrazone (FCCP), which was added to sample wells at the point denoted by the dashed blue lines (re-produced from Fig. 3; *N* = 19; approximately 20 day-1-adult animals per well).

To address this problem, we developed a *C. elegans* respirometry assay using low-cost commercially available optical oxygen sensors and fluorescence plate readers. Our assay utilizes OxoPlates^®^ (manufactured by PreSens Precision Sensing), 96-well microplates featuring phosphorescent oxygen sensors. These systems have been applied to high-throughput investigations of oxygen consumption in bacteria, yeast, and mammalian cell culture, often as a metric of cell viability [30–33], but have seen limited and variable usage in *C. elegans* [34, 35]. Each OxoPlate sensor consists of two dyes embedded in a thin polymer matrix: an indicator dye, whose phosphorescence intensity (*I*_indicator_) is inversely proportional to the oxygen concentration; and a reference dye (*I*_reference_), whose phosphorescence intensity is independent of the oxygen concentration (Fig. 2A). Oxygen concentrations are calculated from internally referenced sensor responses ($I_{R}=\;\frac{I_{\mathrm{indicator}}}{I_{\mathrm{reference}}})$, using *I*_R_ signals derived from oxygen-saturated (*k*_100_) and oxygen-free (*k*_0_) calibration solutions, where $[O_{2}]\;(\%\;\mathrm{air}\;\mathrm{saturation})=100*\frac{\left(\frac{k_{0}}{I_{R}}-1\right)}{\left(\frac{k_{0}}{k_{100}}-1\right)}$. The only hardware requirement for these systems is a plate reader capable of performing fluorescence intensity or, ideally, time-resolved fluorescence measurements, using dual kinetics and bottom optical sensors.

The assay described here was developed for BMG FLUOstar fluorescence plate readers but could likely be adapted for other readers with similar functionality. Our assay utilizes the “kinetic windows” program of the BMG FLUOstar to perform sequential measurements of the *C. elegans* OCR in multiple windows of measurement (oxygen consumption) and linear shaking (re-oxygenation). We describe a simple script to loop several of these programs in sequence with the option of pausing the script for the manual addition of respiratory inhibitors or other interventions, and an accompanying data analysis script to automatically calculate OCRs from linear regressions fit to each measurement window (represented graphically in Fig. 2B and C).

To validate this assay, we first measured basal OCRs in wells with highly variable numbers of day-1-adult stage animals to establish a range of qualitative accuracy, and proceeded to examine the effects of two-well characterized respiratory inhibitors in a set of titration experiments: carbonyl cyanide 4-(trifluoromethoxy) phenylhydrazone (FCCP; a mitochondrial uncoupler [13]) and sodium azide (a Complex IV inhibitor [36]). We then tested whether these drugs can be used sequentially, as standard in intact cell respirometry [13], using optimal concentrations derived from our titration experiments. We show that this assay can be used to accurately estimate the *C. elegans* OCR at a similar scale to Seahorse XF24 and XF96 respirometers (3–72 animals per well) and can capture the effects of FCCP and sodium azide with optimal concentrations highly similar to those reported in Seahorse XF analyser-based studies of *C. elegans* (25 µM FCCP, 24 mM sodium azide). We also show that treatment with FCCP leads to an elevation of the sodium azide-treated (non-mitochondrial) OCR, supporting a previous Seahorse XF analyser study [18] and indicating that accurate measurements of the *C. elegans* non-mitochondrial OCR must be obtained separately from measurements of the maximal OCR.

## Materials and methods

### *Caenorhabditis elegans* strains and maintenance

Bristol N2 (wild type) *C. elegans* were obtained from the Caenorhabditis Genetics Centre (CGC, University of Minnesota). Animals were maintained on Nematode Growth Media (NGM) plates seeded with a thin lawn of *Escherichia coli* OP50 as previously described [37]. Age-synchronized populations were obtained via sodium hypochlorite-based bleaching protocols followed by overnight incubation in M9 at 20°C with gentle rocking [38]. Synchronized L1s were transferred onto NGM plates seeded with *E. coli* OP50 and were grown at 20°C until day 1 of adulthood (72 h later) for all experiments.

### BMG FLUOstar and OxoPlate preparation

All experiments were conducted using a BMG FLUOstar Omega plate reader at 25°C. OxoPlate sensor readouts were obtained using dual kinetic time-resolved fluorescence protocols set up in accordance with PreSens’ documentation. Indicator-dye fluorescence (*I*_indicator_) was measured with an excitation wavelength of 544 nm and an emission wavelength of 655 nm; reference-dye fluorescence (*I*_reference_) was measured with an excitation wavelength of 544 nm and an emission wavelength of 590 nm (see Supplementary Table S1 for additional hardware, software and reagent information). Additional protocol parameters are described in Table 1.

**Table 1.** Basic time-resolved fluorescence protocol parameters.

Multichromatics	Integration time	Settling time	Flashes well^−1^
544 ex/590 em (indicator) 544 ex/655 em (reference)	12–512 µs	0.3 s	20

**Figure 3:**
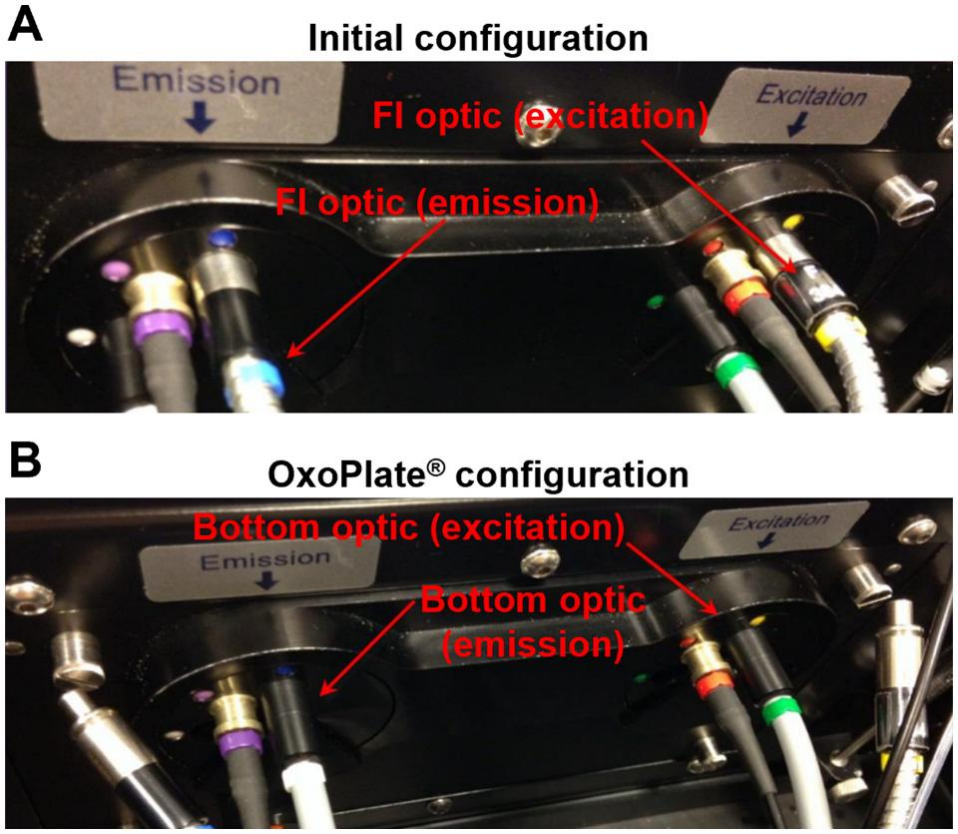
Light guide configuration for bottom-optic time-resolved fluorescence measurements using the BMG FLUOstar. (A) Default light guide configuration with blue and yellow fluorescence intensity (FI) light guides. (B) OxoPlate-compatible configuration in which the yellow and blue FI light guides have been replaced with the white and green bottom-optic light guides, leaving the FI light guides disconnected. Both Images were provided by PreSens.

In its default configuration, the BMG FLUOstar cannot perform time-resolved fluorescence measurements using its bottom optical sensors, which is a requirement for accurately reading the OxoPlate sensors. As such, prior to all experiments the light guides were reconfigured as shown in Fig. 3 in accordance with PreSens’ instructions.

All experiments were conducted using freshly opened OxoPlates or the unused wells of previously opened OxoPlates that were maintained in accordance with PreSens’ instructions. At least 20 min prior to an assay, the OxoPlate sensors were hydrated with a small volume of M9 (80 µl for titration and sequential drug treatment experiments; 20–95 µl for sample size experiments).

### OxoPlate sensor calibration

Each batch of OxoPlates was calibrated using an oxygen-saturated calibration solution (Cal100) and an oxygen-free calibration solution (Cal0). The Cal100 solution was prepared by vigorously shaking Milli-Q water for 2 min in a container with a large air phase (20 ml Milli-Q water in a 50 ml Falcon™ tube). This solution was then rested, uncapped, for 1 min prior to use. The Cal0 solution was prepared as a 1% w/v solution of sodium sulfite in a container with a minimal air phase. These solutions were transferred in 100 µl aliquots to the OxoPlate wells in technical quadruplicate, and the wells containing Cal0 solution were sealed with 100 µl mineral oil to minimize oxygen ingress.

Calibration measurements were preceded by a 30-min incubation in the plate reader to ensure the contents of the wells had equilibrated to the internal temperature of the reader. In accordance with PreSens’ documentation, one calibration was used for all subsequent experiments using the same batch of OxoPlates. To verify whether the media, solvents and drugs used in our validation experiments required separate calibrations, we performed separate calibrations in M9 buffer, with 0.2% dimethylsulfoxide (DMSO; the FCCP solvent), and with optimal concentrations of respiratory inhibitors derived from titration experiments. However, we noted negligible and non-significant alterations to *I*_R_ signals in all these conditions (Supplementary Table S2). As such, all presented oxygen concentrations were calculated using water-based calibrations as described above.

### Sample preparation and loading

*C. elegans* sample preparation followed a modified version of previously published *C. elegans* respirometry protocols [19, 20]. Populations of approximately 300–500 age-synchronized day 1-adult stage *C. elegans* were washed from NGM plates into 15 ml falcon tubes with M9, allowed to settle without centrifugation and the supernatant was discarded. The samples were then washed with M9 and placed on a rocker for 10 min to allow the animals to clear their guts of residual bacteria. Subsequently, the samples were allowed to settle without centrifugation and washed with M9. This was repeated at least twice or until all visible eggs, offspring and bacterial debris had been cleared. After the final wash, the population density of the solution was estimated by calculating the average number of animals in five 20 µl droplets using a dissecting microscope. The solution was then adjusted to an approximate population density of one animal µl^−1^ with M9 and transferred to the hydrated OxoPlate wells to a final well volume of 100 µl. Approximately 20 animals (i.e. 20 µl of solution) were transferred into each well for all experiments with respiratory inhibitors. Sample size experiments used highly variable numbers of animals and adjusted the amount of worm solution accordingly (5–80 µl); for measurements with 80 + animals, the worm solution was concentrated to two to three worms µl^−1^ and transferred in 80 µl aliquots.

### Drug preparation and treatments

Carbonyl cyanide 4-(trifluoromethoxy) phenylhydrazone (FCCP; CAS 370-86-5, Sigma Aldrich) stocks were prepared in DMSO at 500 X final concentration. Sodium azide (CAS 26628-22-8, Sigma Aldrich) stocks were prepared in Milli-Q water at 33 X final concentration.

### OCR measurement protocols and script

OCRs were measured by tracking oxygen consumption over multiple intervals using the BMG FLUOstar’s kinetic windows program. The basic OCR protocol featured four kinetic windows, with readings taken every 20 s for 100 s, each preceded by a 1-min period of linear shaking at 700 RPM to oxygenate the measurement wells (Table 2).

**Table 2.** Time-resolved fluorescence protocol parameters for oxygen consumption rate measurements.

Multichromatics	Integration time	Settling time	Flashes well^−1^	Kinetic windows	Cycle time	Cycles	Linear shaking (700 RPM)
544 ex/590 em (indicator) 544 ex/655 em (reference)	12–512 µs	0.3 s	20	4	20 s	6	1 min before each window

With these protocol parameters, maintaining a cycle time of 20 s allowed for the measurement of 10 wells per protocol, and brought the length of each protocol to a total of 12 min.

This protocol was then looped several times in sequence using the following BMG FLUOstar script:

1. st1: = “OxoPlate protocol” #Pre-configured time-resolved fluorescence OxoPlate protocol

2. NumberOfReadings: = n #Number of protocol loops

3. wait for 10 min #Temperature equilibration period

4. for i = 1 to NumberOfReadings do begin

5.     ID1:=“Reading ” i

6.     R_run “<st1>”

7. If i = 3 Then begin

8.     Ask “Add drug A and click Yes to continue “ (“Continue”)

9. End

10. If i = 6 Then begin

11.     Ask “Add drug B and click Yes to continue “ (“Continue”)

12.     End

13. Next i

Pauses were implemented every three cycles to eject the plate from the reader and treat the sample wells with respiratory inhibitors. As the aqueous solubility of oxygen decreases with temperature [39], an initial wait period was included to ensure the temperature of the wells had equilibrated to the internal temperature of the plate reader. The length of this period was derived by measuring the oxygen concentration of Milli-Q water following a shift from 20°C (laboratory temperature) to 25°C (the internal temperature of the plate reader), which appeared to stabilize approximately 10 min following the temperature shift (Supplementary Fig. S1).

For each assay, the number of protocol loops (*n*) were altered, with sample-size tests assessed with two (total reading time: approximately 34 min), drug titrations assessed with six (three control and three drug-treated; total reading time: approximately 82 min), and sequential drug treatment experiments assessed with nine (three control and six drug treated; total reading time: approximately 108 min).

Following each assay, the contents of each well were extracted using pipette tips coated in 0.01% Triton X-100 in M9, and the number of animals residing in each well was counted by eye using a dissecting microscope.

### Data analysis and statistics

Internally referenced (*I*_R_) sensor responses were calculated from reference- and indicator-dye phosphorescence using the BMG MARS data analysis software, where $I_{R}=\frac{655_{\mathrm{em}}}{590_{\mathrm{em}}}$. Oxygen concentrations were calculated using calibration constants derived from the average I_R_ signals of oxygen-free (*k*_0_) and oxygen-saturated (*k*_100_) calibration solutions (see OxoPlate sensor calibration), where: $[O_{2}]\;(\%\;\mathrm{air}\;\mathrm{sat}.)=100*\frac{\left(\frac{k_{0}}{I_{R}}-1\right)}{\left(\frac{k_{0}}{k_{100}}-1\right)}$.

OCRs were quantified in each kinetic window by fitting linear regressions to the first minute of oxygen consumption, extracting the regression coefficient, and multiplying the result by −1, i.e. assuming $[O_{2}]\approx \mathrm{at}+b,\;\mathrm{OCR}\;\approx -a.\;$OCRs were then normalized to the number of worms within each well. This process was automated using a custom R script (see Automated OCR calculation script in the Supplementary Information; for the required formatting of *I*_R_ data, see Supplementary Fig. S2).

Parameters of respiratory function were calculated as follows: basal (i.e. routine or untreated) OCRs were calculated as the average OCR prior to treatment with any respiratory inhibitor; maximal OCRs were calculated as the average of the highest three OCRs following treatment with FCCP, with the first three measurement windows following FCCP treatment excluded; and non-mitochondrial OCRs were calculated as the average OCR following treatment with sodium azide.

All statistics were calculated using R v. 4.5.6. The effects of sample size on per-well OCRs were analysed via linear regression. Unless stated otherwise, all remaining data were analysed using ANOVA with *post hoc* Tukey’s Honestly Significant Differences (TukeyHSD) tests (see the Data handling and statistical analysis script in the Supplementary Information for further details). All graphs were produced using the R package *ggplot2* [40] and all data presented therein represent the mean ± standard error.

## Results and discussion

### Sample size analysis

**Figure 4:**
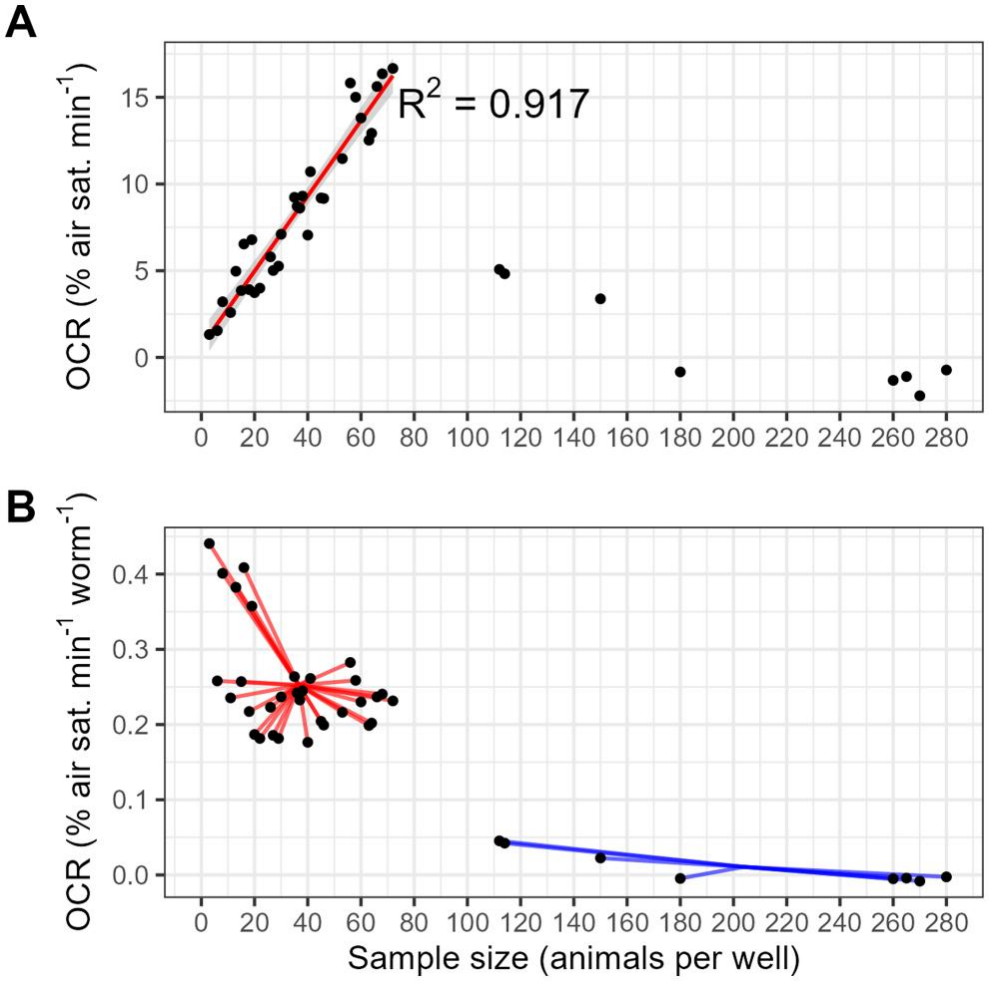
The impact of sample size on basal OCRs. (A), Oxygen consumption rates (OCRs) in wells containing 3–280 day-1-adult *C. elegans*. The linear regression is fitted to OCR data from 3—72 animals per well. (B), Per-animal OCRs in wells containing 3—280 animals. The line segments within each group are drawn from each data point to the group-mean OCR (mean OCR *N* = 3–72: 0.248% air sat. min^−1^ worm^−1^; mean OCR *N* = 112–280: 0.011% air sat. min^−1^ worm^−1^). *N* = 40.

To validate our assay, we first examined the relationship between per-well OCRs and sample size, as a linear relationship between sample size and oxygen consumption would establish a range of comparative accuracy for subsequent validation experiments. Therefore, we measured OCRs in wells containing highly variable numbers of animals (3–280 per well). Our results indicated that per-well OCRs were linear with sample size between 3 and 72 animals per well (*R*^2^ = 0.92, *F*_1, 30_ = 334.5, *P* < .0001; Fig. 4A), with an average per-animal OCR of 0.248% air sat. min^−1^ worm^−1^. OCRs sharply declined beyond this range (112–280 animals per well), with an average per-animal OCR of 0.011% air sat. min^−1^ worm^−1^ (*P* < .0001 relative to per-animal OCRs in the linear range, unpaired *t*-test; Fig. 4B), as the high rate of oxygen consumption exceeded the capacity of the linear shaking period to re-oxygenate the wells (Supplementary Fig. S3A), resulting in OCR-limiting hypoxia (Supplementary Fig. S3B).These results demonstrate that this assay can be used to estimate the *C. elegans* OCR with relatively small quantities of worms, making sample preparation straightforward. This requirement for biological material (3–72 day 1 adult-stage animals per well) is similar to the amount required for Seahorse XF96 and XFp respirometers (∼2–25 day 1 adult-stage animals per well [20]) and Seahorse XF24 respirometers (∼50 day 1 adult-stage animals per well [23]), and far lower than the requirement for Clark-type respirometry (300–1000 day 1 adult-stage animals per chamber [24, 41]).

### OCR responses to respiratory inhibitors

Next, we tested whether our assay could capture alterations in respiratory activity caused by respiratory inhibitors with documented efficacy in *C. elegans*. Respirometry in intact cells is typically performed with the mitochondrial ATP synthase inhibitor oligomycin to assess ATP synthase-coupled respiration, the protonophore FCCP to assess maximal (uncoupled) respiration, and a combination of the Complex I inhibitor rotenone and Complex III inhibitor antimycin A to assess non-mitochondrial respiration [14]. However, with the exception of FCCP, these compounds are ineffective in *C. elegans*, likely due to poor diffusion through the cuticle [18, 20]. Instead, respirometry experiments in *C. elegans* generally use FCCP to assess maximal respiration, and the Complex IV inhibitor sodium azide to assess non-mitochondrial respiration [18–22]. The non-specific ATP synthase inhibitor dicyclohexylcarbodiimide (DCC) [42] has also been used to examine mitochondrial ATP synthase-linked respiration in *C. elegans* [18, 19, 43], but its activity requires a significant incubation period [18], and at least one study was unable to observe any response following treatment of *C. elegans* with DCC [21]. We therefore focused primarily on examining the effects of FCCP and sodium azide.

To determine the optimal concentrations of these respiratory inhibitors, we performed a set of titration experiments. Concentrations of 25 µM FCCP and 12 mM sodium azide were taken as starting points based on similar estimates of optimal concentrations reported in Luz *et al.* [19], and a range of concentrations around these values, in log_2_ scale, were examined, with OCR responses assessed following a period of basal (untreated) respiration. Based on our previous sample size results, and anticipating the eventual sequential use of FCCP and sodium azide, we opted to use 20 animals per well for all subsequent experiments, reasoning that this would allow OCRs to fluctuate significantly while remaining within the linear OCR range shown in Fig. 4A.

**Figure 5:**
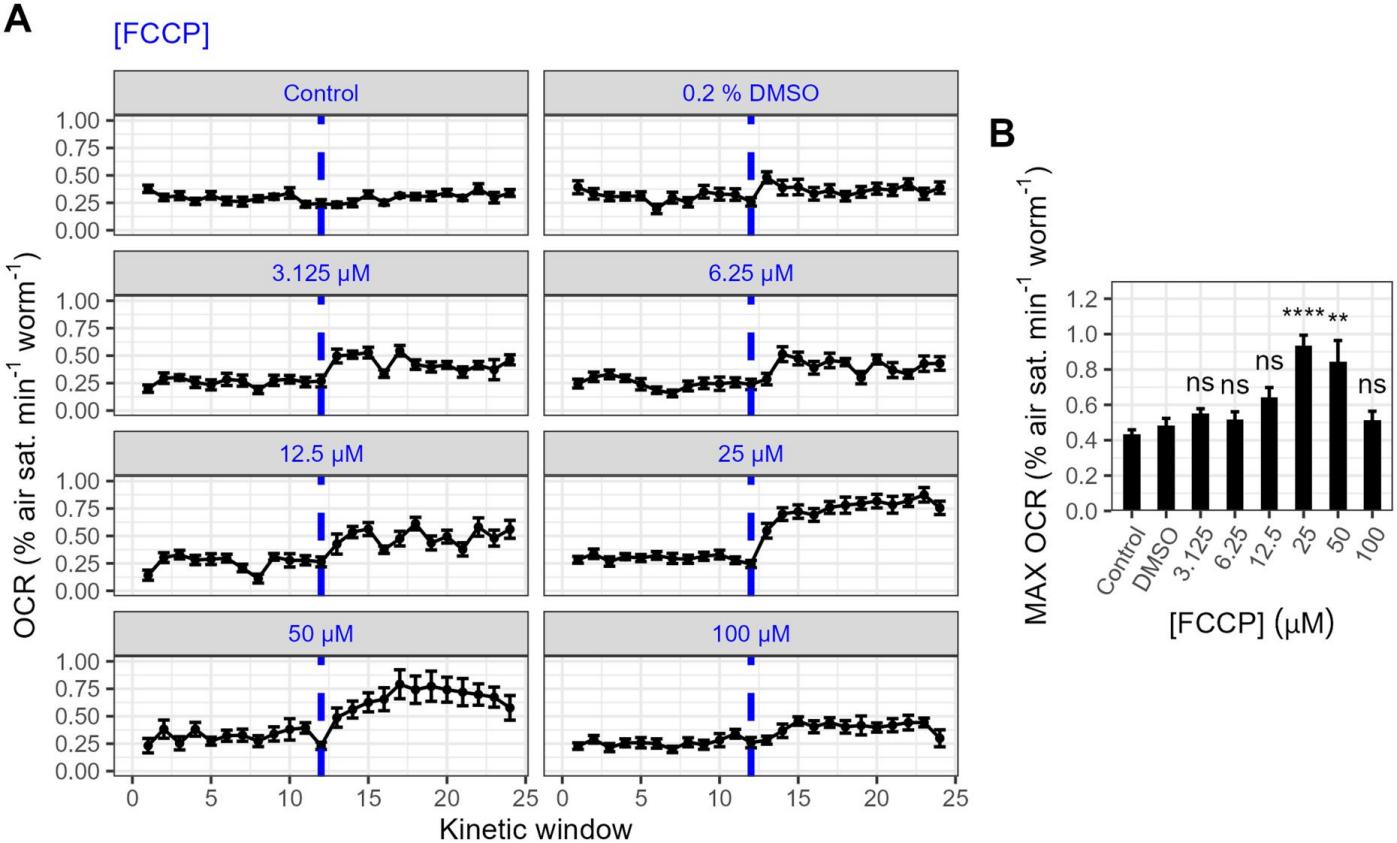
The effects of FCCP on the *C. elegans* OCR. (A) Oxygen consumption rate (OCR) traces of *C. elegans* samples treated with various concentrations of FCCP (dashed lines). Untreated control samples received no drug or solvent treatment, and solvent controls received an injection of DMSO (final concentration 0.2% v/v). **(B)** Maximal OCRs calculated from OCR traces presented in A. Maximal OCRs were calculated as the average of the highest three OCR measurements within kinetic windows 16–24. ****P < .0001; **P < .01; ns, not significant (*P* > .05), TukeyHSD test relative to DMSO controls. *N* = 9–19; approximately 20 day-1-adult *C. elegans* per well.

As expected, treatment with FCCP significantly altered the maximal respiration rate (*F*_7, 84_ = 12.1, *P* < .0001; Fig. 5A), with a clear peak response observed at 25 µM (Fig. 5B). Treatment with 50 µM FCCP did not further alter the maximal OCR relative to the 25 µM treatment (*P* = .945; TukeyHSD), while treatment with 100 µM FCCP induced a significant decline (*P* < .0001; TukeyHSD). As FCCP is water insoluble, it is possible that this was partially caused by the precipitation of FCCP over the course of the assay. However, studies in intact cells have demonstrated that excessive concentrations of protonophores such as FCCP can lead to a severe drop in respiratory activity, likely resulting from a reduction in substrate import caused by the collapse of the proton-motive force [44], which may have contributed to the repression of maximal OCRs observed here. Our estimated optimal concentration of 25 µM matches that described in Luz *et al.* [19] and is within the range described in *C. elegans* Seahorse respirometry protocols, which commonly use concentrations between 10 and 25 µM [19–23]. Moreover, the responses we found here are of approximately the same relative magnitude (1.92-fold increase relative to DMSO controls at 25 µM FCCP) as those reported elsewhere (∼2-fold increase relative to DMSO controls in Koopman *et al.* [20] and Luz *et al.* [18]), indicating that our assay is able to accurately capture maximal respiration in *C. elegans*.

**Figure 6:**
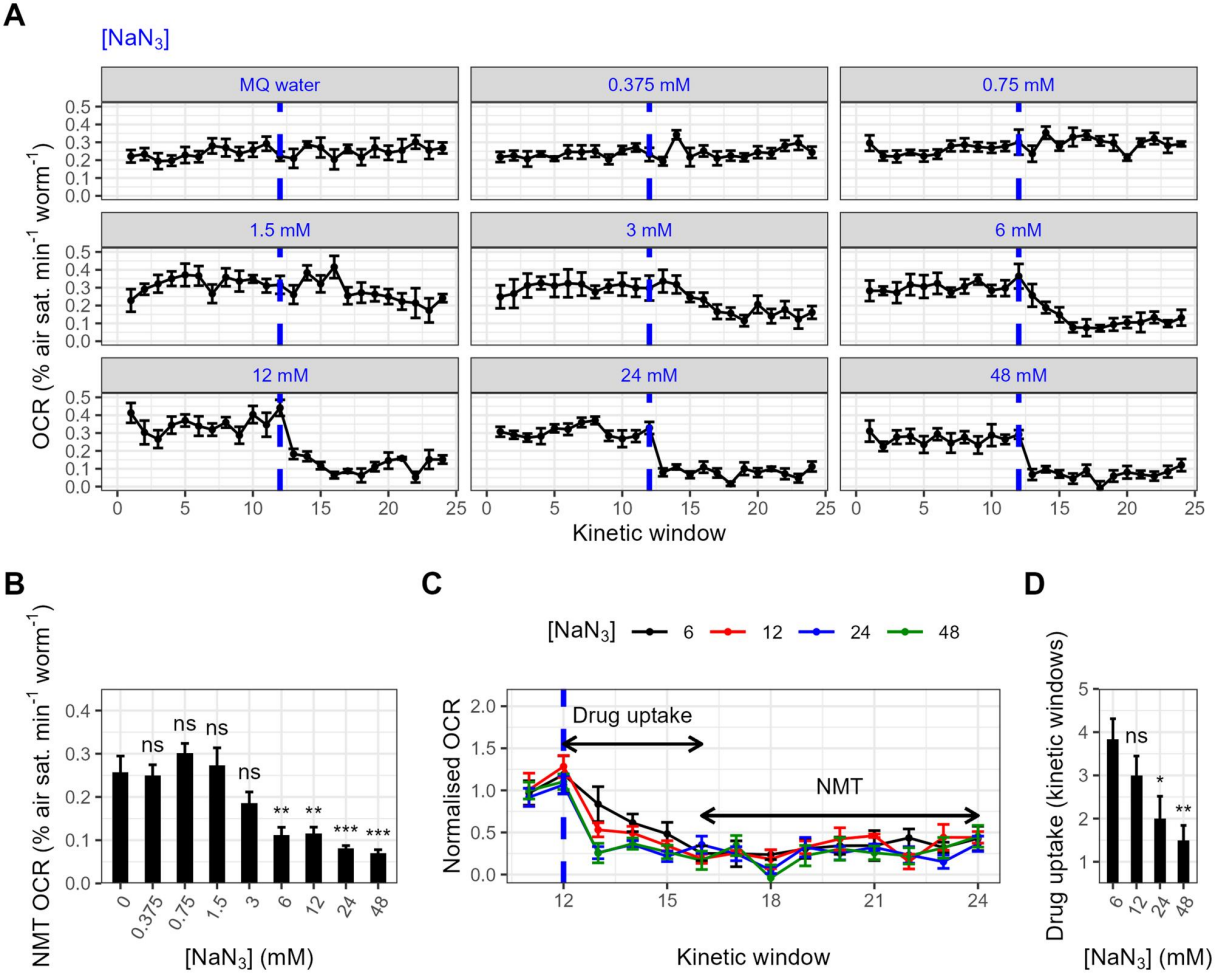
The effects of sodium azide on the *C. elegans* OCR. (A) Oxygen consumption rate (OCR) traces of *C. elegans* samples treated with various concentrations of sodium azide (NaN_3_; dashed lines). Solvent controls received an injection of Milli-Q (MQ) water. (B) Non-mitochondrial (NMT) OCRs calculated from the OCR traces presented in A. Non-mitochondrial OCRs were calculated as the average of all OCR measurements within kinetic windows 13–24. ***P < .001; **P < .01; ns, not significant (*P* > .05), TukeyHSD test relative to MQ water controls. (C-D) Basal-normalized oxygen consumption rate (OCR) of samples treated with 6–48 mM sodium azide (NaN_3_; dashed line), highlighting concentration-dependent differences in drug uptake. OCRs were normalized using the sample-mean basal OCR (kinetic windows 1–12). B, Average drug uptake time, defined as the number of measurements (kinetic windows) required to reach the pooled average basal-normalized non-mitochondrial OCR (0.287). **P < .01; *P < .05; ns, not significant (*P* > .05), TukeyHSD test relative to 6 mM-treated samples. *N* = 5–7; approximately 20 day-1-adult *C. elegans* per well.

Treatment with sodium azide also strongly affected OCRs (*F*_8, 44_ = 14.15, *P* < .0001; Fig. 6A), with significant reductions observed at concentrations greater than 6 mM (Fig. 6B). Relative to the 6 mM treatment, higher concentrations of sodium azide were not associated with further repression of the OCR (all *P* > .938; TukeyHSD), but were associated with a shorter uptake time (the number of kinetic windows required to reach the average basal-normalized non-mitochondrial OCR; *F*_3, 20_ = 5.34, *P* = .007; Fig. 6C), with the fastest responses observed in the 24 mM and 48 mM treatments (Fig. 6D). As the 48 mM treatment did not further reduce the uptake time relative to the 24 mM treatment (*P* = .860; TukeyHSD), we concluded that the optimal concentration of sodium azide was 24 mM. This concentration is within the range described in *C. elegans* Seahorse respirometry protocols, which commonly use concentrations between 10 mM and 50 mM [19–22]. Like our FCCP data, our results are also qualitatively comparable to those reported in other studies. With the optimal concentration of sodium azide (24 mM), OCRs were reduced to 24.2% of MQ water controls, compared to approximately 28.5% in Luz *et al.* [16], approximately 14.3% in Koopman *et al.* [20] and approximately 20% in Ng and Gruber [21], indicating that our assay is able to accurately capture non-mitochondrial oxygen consumption in *C. elegans*.

### The effects of FCCP on the sodium azide-treated OCR

Respirometry experiments involving intact cells are typically performed with the sequential addition of respiratory inhibitors [13], as this allows multiple parameters of respiratory function, such as ATP synthase-linked respiration, maximal respiration, and non-mitochondrial respiration, to be assessed on a per-sample basis. However, this may not be advisable in *C. elegans*, as early *C. elegans* Seahorse respirometry studies reported that the sequential use of FCCP and sodium azide increased the non-mitochondrial (sodium azide-treated) OCR relative to estimates derived using sodium azide alone [18], though a later attempt to replicate this result found no such difference [20]. Given this ambiguity, we sought to determine whether non-mitochondrial OCRs were similarly affected by prior FCCP supplementation using our assay. Therefore, we tracked OCRs in *C. elegans* samples treated with 24 mM sodium azide following prior treatment with 25 µM FCCP, prior treatment with 0.2% v/v DMSO (solvent controls), or no prior treatment (untreated controls).

**Figure 7:**
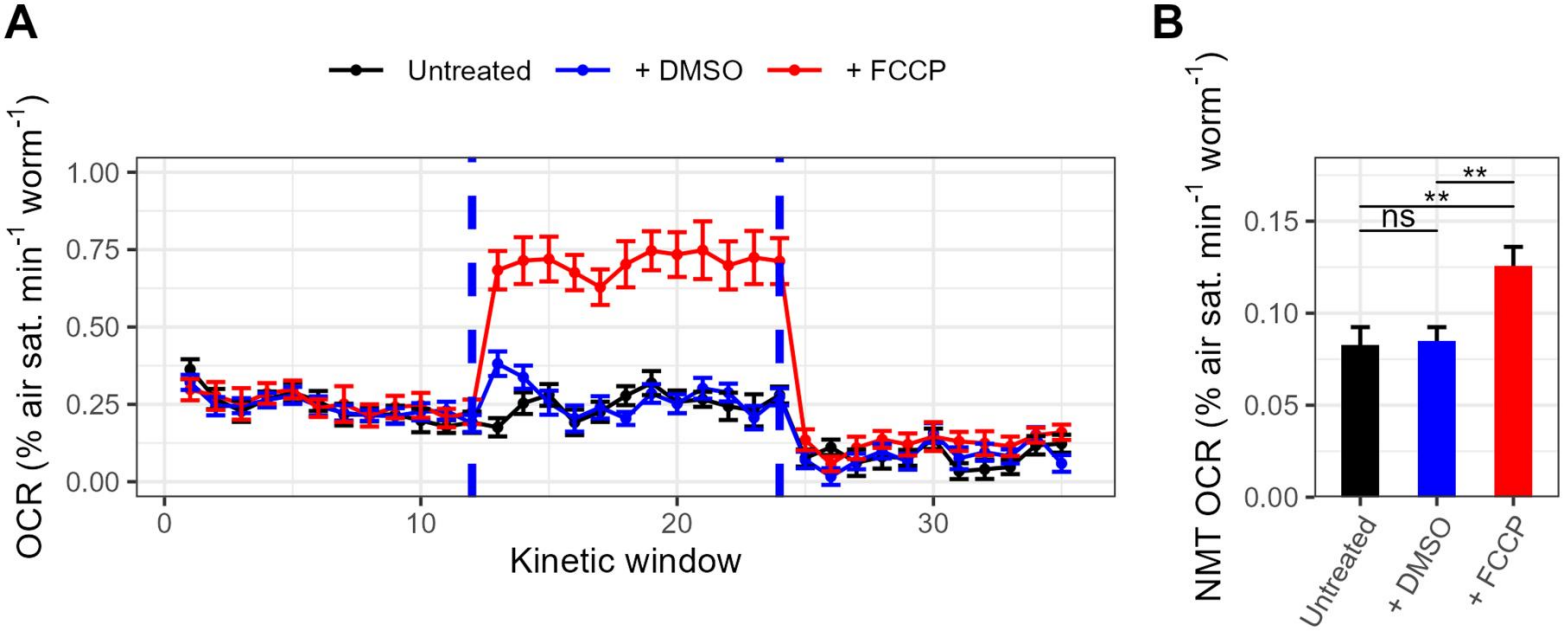
The effects of sodium azide post-FCCP. (A) Oxygen consumption rate (OCR) traces of animals treated with sodium azide following prior treatment with 25 μM FCCP, prior treatment with 0.2% v/v DMSO, or no prior treatment. FCCP and sodium azide treatments are denoted by the dashed lines. (B) Non-mitochondrial (NMT) OCRs of untreated, DMSO-treated and FCCP-treated samples. NMT OCRs were calculated as the average OCR within kinetic windows 25–35. **P < .01; ns, not significant (*P* > .05), TukeyHSD test. *N* = 15–17; approximately 20 day-1-adult *C. elegans* per well.

Our results indicated that the sodium azide-treated OCRs were affected by prior treatment (*F*_2, 44_ = 6.9, *P* = .003), with OCRs significantly elevated in the FCCP-treatment group relative to both untreated and solvent controls (Fig. 7A and B). DMSO did not alter the sodium azide-treated OCR relative to untreated controls (*P* = .979, TukeyHSD), suggesting that FCCP alone impaired the effects of sodium azide. Therefore, our results suggest that assessing the non-mitochondrial OCR with sodium azide immediately following FCCP treatment will lead to an over-estimation of non-mitochondrial oxygen consumption and thus should be avoided when performing respirometry in *C. elegans*.

### General comparisons with standard methods of *C. elegans* respirometry

The assay described here allows for cost-effective, repeated OCR measurements of *C. elegans* samples with a moderate throughput. In general outline this assay resembles Seahorse XF analyser-based respirometry; oxygen levels are measured using phosphorescent oxygen sensors in a 96-well plate format, with OCRs quantified over multiple periods of oxygen consumption (measuring) and re-oxygenation (shaking). However, the major strength of this assay relative to typical high-throughput microplate-based respirometry systems is that it is comparatively inexpensive, particularly on a per-assay basis [29]. At the time of purchase (January 2022), a batch of 50 OxoPlates cost £597, giving a per plate cost of £11.94 and a per sample cost of ∼12p (assuming all 96 wells are used). However, total costs will likely vary for prospective users due to shifts in reagent pricing and discounts associated with the bulk purchasing of plates.

This assay was developed without the use of a mineral oil or adhesive covering to minimize oxygen ingress, as it was necessary to keep the wells open for re-oxygenation following each measurement period. As such, OCRs calculated using this assay are offset by oxygen diffusion from the environment, which worsens during periods of high oxygen consumption (Supplementary Fig. S4A and B). High rates of oxygen consumption, such as during FCCP exposure, may also outstrip the capacity of the shaking period to re-oxygenate the wells, depleting the baseline oxygen concentration and leading to an under-estimation of the OCR. However, issues related to re-oxygenation of the sample wells can be mitigated by working within a linear OCR range as described above and reducing the sample size appropriately. Seahorse XF respirometers are also open systems and experience significant oxygen diffusion [13], but obtain quantitatively accurate OCRs by correcting raw [O_2_] data for oxygen ingress using an algorithm which employs calibration data from Clark-type respirometers [45]. We did not develop any method for the *post hoc* correction of our [O_2_] data, and instead minimized the issue of oxygen diffusion by calculating OCRs over a relatively short period (60 s) in comparison to the standard 2–3 min period used by Seahorse XF analysers [13] (Supplementary Fig. S4B and C). This improved the goodness-of-fit of linear regressions during periods of high (maximal) oxygen consumption relative to those obtained over the full 100 s kinetic window (Supplementary Fig. S4D), but did not eliminate differences in goodness-of-fit between high (maximal) and low (basal and non-mitochondrial) oxygen consumption. Therefore, OCRs calculated using this assay may not be quantitatively accurate due to oxygen diffusion and, consequently, we have made no quantitative comparisons of our OCR data to those derived via other methods. However, our validation experiments strongly suggest OCRs calculated using this assay remain reproducible for comparative analyses.

Since our sequential drug treatment experiments suggest that FCCP cannot be used in conjunction with sodium azide, we anticipate that others wishing to make use of this assay will likely follow the same structure used in our titration experiments, with a total assay length of approximately 82 min. Sample preparation and other pre-assay steps, which took no more than one hour, brought the total length of these experiments to approximately 2–2.5 h, comparable to the length of published *C. elegans* microplate-based respirometry protocols [19–23], and easily suitable for multiple experiments in a single work day.

While maintaining a constant number of measurements (six) and measurement interval (20 s), this assay allows for the measurement of a total of 10 samples per run. This throughput is similar to the 8-well Seahorse XFp [22], and significantly higher than the two-chamber Oxygraph-2k [29], but lower than the 24-well Seahorse XF24 [18, 19] and 96-well XF96 [20]. However, pooling data from replicate Seahorse experiments in *C. elegans* is not advised in published protocols due to their fixed 37°C heating element [20]. As this would represent an acute heat stress for *C. elegans* [46], this heating element must be deactivated prior to use, allowing the internal temperature to equilibrate to laboratory temperature but leaving it free to increase by as much as 5°C during operation [19–23]. In contrast, the BMG FLUOstar’s modifiable heating element allowed us to maintain an internal temperature of 25°C (its lowest setting), which typically deviated by <1°C during an experiment. While even a small elevation in temperature such as this is likely to alter metabolic rates [47, 48], we argue that if short-term alterations in temperature are predicted to significantly alter the *C. elegans* OCR, then it is better to maintain a constant elevated temperature than to allow temperatures to slowly increase over the course of an experiment, possibly leading to erroneous estimates of respiratory parameters calculated from OCRs at different ambient temperatures. A constant incubation temperature, combined with the inherent reduction of technical variability related to the internal referencing of the OxoPlate sensors, enabled us to pool data from multiple runs for analysis, thereby making the practical throughput of the assay similar to the Seahorse XF24 and XF96 analysers, and thus making it suitable for larger experiments with multiple conditions.

### Conclusions

Here we describe the development and validation of a cost-effective assay for *C. elegans* respirometry which does not require access to specialized respirometry equipment. We validate our assay for comparative accuracy in day 1 adults by demonstrating the linearity of basal OCRs in samples with highly variable numbers of animals, and by examining the effects of the respiratory inhibitors FCCP and sodium azide in titration experiments. We then apply our assay to demonstrate that prior treatment with FCCP elevates sodium azide-treated OCRs, supporting previous *C. elegans* respirometry data, and strongly suggesting that estimates of the maximal and non-mitochondrial OCR must be obtained separately when working with *C. elegans*.

While our experiments were limited to wild-type day 1 adults, this assay could be easily adapted for use in animals of different ages or genetic backgrounds by simply replicating our sample size and titration experiments. This assay will be useful for researchers who wish to perform respirometry in *C. elegans* in a cost- and time-efficient manner.

## Supplementary Material

bpaf072_Supplementary_Data

## Data Availability

Data available on request.
