# Supplementary material for: A novel C. elegans respirometry assay using low-cost optical oxygen sensors
Source: Biol Methods Protoc. 2025 Sep 30;10(1):bpaf072. doi: 10.1093/biomethods/bpaf072 (PMC12557035; doi:10.1093/biomethods/bpaf072)
Supplement: bpaf072_Supplementary_Data [file bpaf072_supplementary_data.zip › Supp. materials OUP submission clean.docx]

Supplementary Information

Automated OCR calculation script

To expedite the calculation of OCRs from internally referenced (I_R_) sensor data, we created a set of functions in R v. 4.5.6 to automatically calculate oxygen concentrations, fit linear regressions within user-defined measurement windows, extract the regression coefficients, multiply the coefficients by -1, and normalise the resultant OCR data using a list of normalisation parameters (here the number of animals per sample well). This script requires that the data be formatted with a single “time” column (which must be in lowercase and formatted in seconds), and multiple I_R_ data columns with the name of the condition specified first, followed by an underscore and a designation for the replicate (see Figure S2). This script also requires user-defined calibration constants derived from oxygen-free (k_0_) and oxygen-saturated (k_100_) calibration solutions (see OxoPlate sensor calibration), and the pre-installation of the *tidyverse* and *forstringr* packages. For the OCR calculation script, the OCR calculation window is set by the interval value (the number of measurements for regression-fitting), and the total length of each kinetic window (as set by the number of cycles in the BMG FLUOstar protocol) is set by the step value.

1. #User-defined calibration constants

2.

3. k0 <- 95.9725

4. k100 <- 31.16333

5.

6. #OCR functions ---------------------------

7.

8. require(tidyverse)

9. require(forstringr)

10.

11. #IR -> O2 function

12.

13. o2.fun <- function(data, k0, k100){

14. df <- cbind(data[,1], 100*(k0/data[,-1]-1)/(k0/k100-1))

15. return(df)

16. }

17.

18. #Function to extract slope of a linear regression

19.

20. get_slope <- function(x, y) {

21. lm_model <- lm(y ~ x)

22. rsquared <- summary(lm_model)$r.squared

23. return(coef(lm_model)[2])

24. }

25.

26. #OCR function

27.

28. OCR.fun <- function(data, interval, step, worm.no) {

29.

30. slopes <- numeric() #Record of OCRs

31. col_names <- character() #Record of sample names

32. interval_numbers <- numeric() #Record of kinetic windows

33. interval_number <- 1

34.

35. for (i in seq(1, nrow(data), by = step)) { #Loop to subset the data for each kinetic window

36. subset_data <- as.data.frame(data[i:(i + interval - 1), ])

37. time_subset <- subset_data$time #Extracts time column for OCR calculation

38.

39. for (col in 2:ncol(subset_data)) { #Loop to fit linear regressions to each sample within the current kinetic window

40. slope <- get_slope(time_subset, subset_data[, col]) #Records the raw OCR

41. slopes <- c(slopes, slope / worm.no[col - 1]) #Records the normalised OCR

42. col_names <- c(col_names, names(subset_data)[col]) #Records the sample name

43. interval_numbers <- c(interval_numbers, interval_number) #Records the kinetic window

44. }

45. interval_number <- interval_number + 1

46. }

47.

48. result <- data.frame(cycle = interval_numbers, #Combines all the data collected into a single data frame in long format

49. rep = col_names, der = -slopes * 60, #Conversion of OCR from per sec. to per min.

50. condition=str_extract_part(col_names, "_", before = T)) #Extracts condition identifier (all characters before "_")

51.

52. return(result)

53. }

Data handling and statistical analysis script

To simplify data handling and analysis downstream of OCR quantification, we developed the following script for the automated calculation of basal, maximal and non-mitochondrial OCRs, and statistical analyses appropriate for sample size and drug titration experiments. The required input for this script is a dataframe produced by the OCR function (see previous section).

1. require(tidyverse)

2. require(rstatix) #For the pipe-friendly TukeyHSD

3. require(forstringr) #For string manipulation

4.

5. state.fun <- function(data, b.window = NA, m.window = NA, nmt.window = NA) { #Function to extract basal, maximal and non-mitochondrial OCRs from OCR traces, and produce a dataframe with a “condition” column (extracted from sample names) which can be used for statistical analysis. Requires a data frame generated by OCR.fun and user-defined ranges of the basal (b.window), maximal (m.window) and/or non-mitochondrial (nmt.window) OCR (in kinetic windows: e.g. b.window = c(1:12)).

6.

7. data.r <- data |>

8. group_by(rep) |>

9. filter(cycle %in% b.window) |>

10. summarise(der = mean(der, na.rm=T)) |>

11. mutate(condition = str_extract_part(rep, "_", before = T),

12. state = "basal")

13.

14. data.m <- data |>

15. group_by(rep) |>

16. filter(cycle %in% m.window) |>

17. arrange(-der) |>

18. summarise(der = mean(head(der, 3), na.rm=T)) |> #Maximal respiration is calculated as the mean of the highest 3 measurements within the m.window

19. mutate(condition = str_extract_part(rep, "_", before = T),

20. state = "max")

21.

22. data.nmt <- data |>

23. group_by(rep) |>

24. filter(cycle %in% nmt.window) |>

25. summarise(der = mean(der, na.rm=T)) |>

26. mutate(condition = str_extract_part(rep, "_", before = T),

27. state = "nmt")

28.

29. return(rbind(data.r, data.m, data.nmt))

30.

31. }

32.

33. summary(lm(der ~ as.numeric(condition), data = dat)) #Linear regression for sample size experiments

34.

35. summary(aov(der ~ factor(condition), data = dat, subset = state == “nmt or max”)) #ANOVA for drug treatment experiments

36.

37. aov(der ~ factor(condition), data = dat) |> #Post-hoc TukeyHSD for drug treatment experiments

38. filter(state == “nmt or max”) |>

39. tukey_hsd()

Supplementary Tables

Table S1. Hardware, software and reagent information.

| Hardware requirements | Additional information |
| --- | --- |
| BMG FLUOStar Omega Filter-based multi-mode microplate reader | Filters: 544 nm (excitation), 590 nm (emission), 655 nm (emission)  Optic setting: Bottom optics  Reading mode: Plate-mode  Method: Time-resolved fluorescence  No. multichromatics: 2 |
| OxoPlate OP96U | 96-well U-bottom plates (Greiner Microlon^®^ 600) |
| Software requirements and recommendations | -- |
| BMG MARS data analysis software | Required for extracting I_R_ data |
| R v. 4.5.6 | Required for OCR and data analysis scripts |
| RStudio | Recommended for data analysis and visualisation |
|  |  |
| Reagent | Source or recipe |
| M9 | 3 g L^-1^ KH_2_PO_4_, 6 g L^-1^ Na_2_HPO_4_, 5 g L^-1^ NaCl, 0.12 g L^-1^ MgSO_4_ |
| FCCP (CAS 370-86-5) | Sigma Aldrich |
| Sodium azide (CAS 26628-22-8) | Sigma Aldrich |

Table S2. Calibration constants for all media, solvent and drug treatments. p values represent the results of FDR-corrected unpaired t-tests relative to Milli-Q (MQ) H_2_O controls. N=3-4.

| Calibration constant | Solvent, media or drug treatment | I_R_ (mean ± SE) | p |
| --- | --- | --- | --- |
| k_0_ | MQ H_2_O | 95.97 ± 0.67 | -- |
|  | M9 | 99.25 ± 0.96 | 0.142 |
|  | 0.2 % v/v DMSO | 97.43 ± 0.47 | 0.204 |
|  | 25 µM FCCP | 97.74 ± 0.62 | 0.204 |
|  | 24 mM sodium azide | 99.03 ± 0.63 | 0.080 |
|  | 25 µM FCCP + 24 mM sodium azide | 97.98 ± 0.28 | 0.154 |
| k_100_ | MQ H_2_O | 31.16 ± 0.18 | -- |
|  | M9 | 30.44 ± 0.43 | 0.776 |
|  | 0.2 % v/v DMSO | 30.98 ± 0.35 | 0.938 |
|  | 25 µM FCCP | 30.65 ± 0.24 | 0.725 |
|  | 24 mM sodium azide | 30.66 ± 0.28 | 0.776 |
|  | 25 µM FCCP + 24 mM sodium azide | 31.35 ± 0.14 | 0.938 |


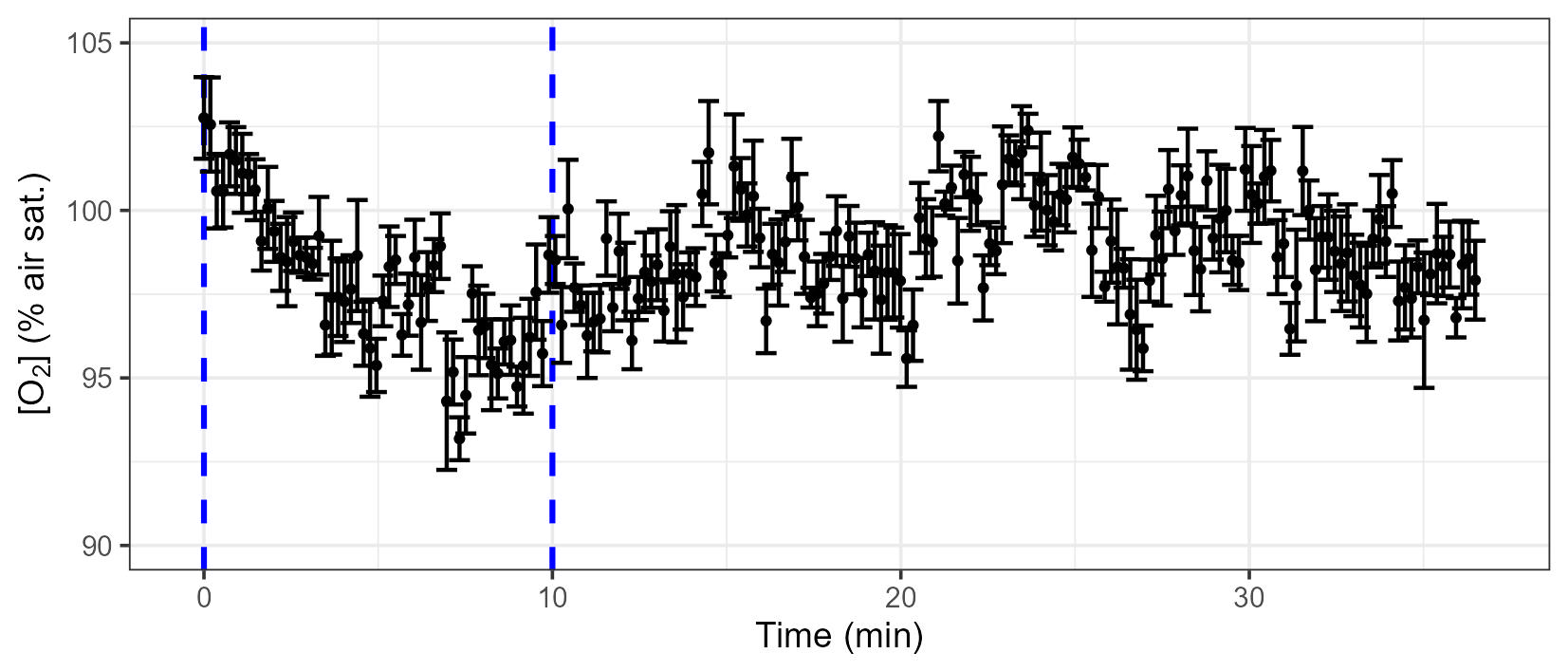
Supplementary Figures

Figure S1. Estimate of temperature equilibration time following a shift from 20 ^o^C to 25 ^o^C. Oxygen levels were calculated based on a two-point calibration at 25 ^o^C. The point at which oxygen solubility ceased declining (approximately 10 minutes following the shift in temperature, dashed lines) was used to approximate the length of time required for the temperature of the sample wells to equilibrate to the internal temperature of the plate reader. N = 5, mean ± SE.


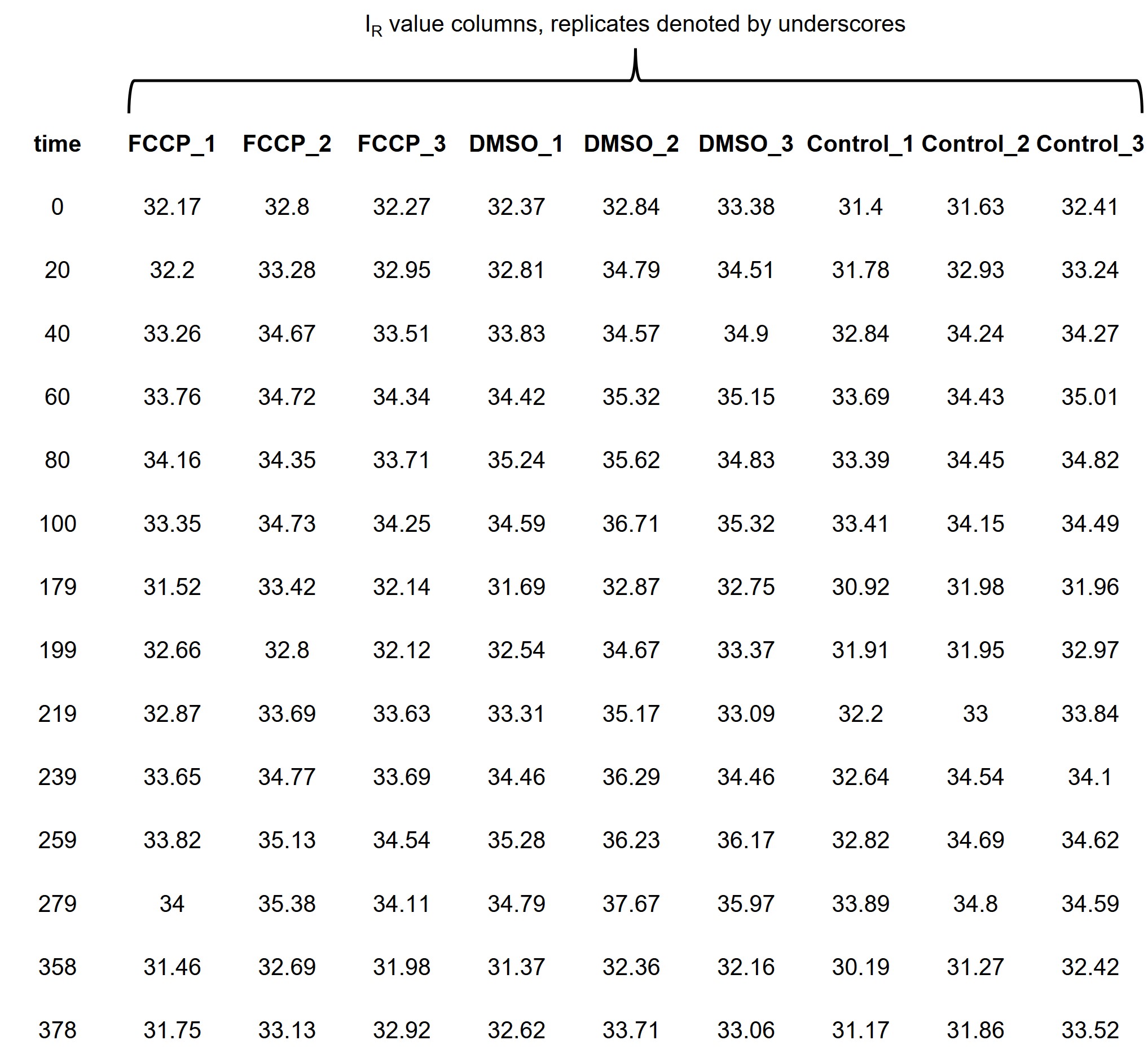


Figure S2. Example I_R_ data table for automatic OCR analysis. The time identifier must be labelled “time” (in lowercase) and formatted in seconds. Each data column must be formatted with the name of the condition first (here FCCP, DMSO or Control) followed by a designation for the replicate (numbers or letters) separated by an underscore, i.e. FCCP_1 refers to the first replicate of the condition “FCCP”.


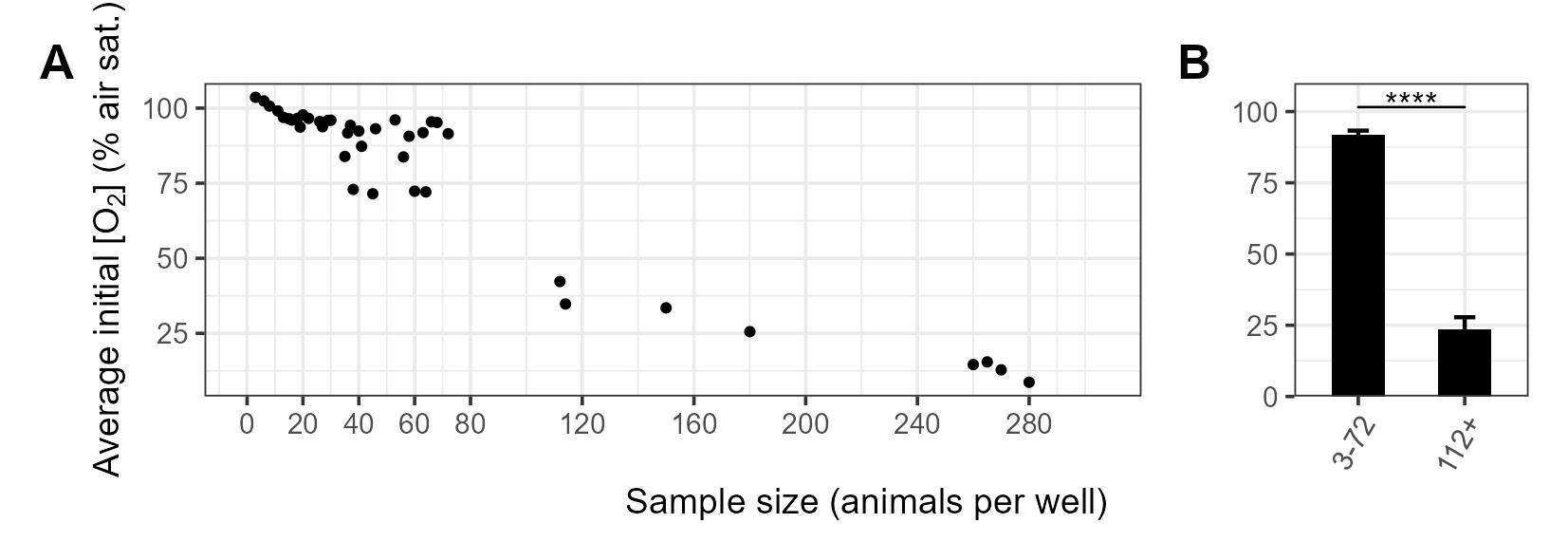


Figure S3. The impact of sample size on average first-measurement oxygen concentrations. (A) Average first-measurement oxygen concentrations in wells containing 3-280 animals. (B) Average first-measurement oxygen concentrations over the linear OCR range (3-72 animals per well) and the non-linear range (112-280 animals per well). N = 40, mean ± SE; approximately 20 day-1-adult *C. elegans* per well. ****, p < 0.0001, unpaired t-test.


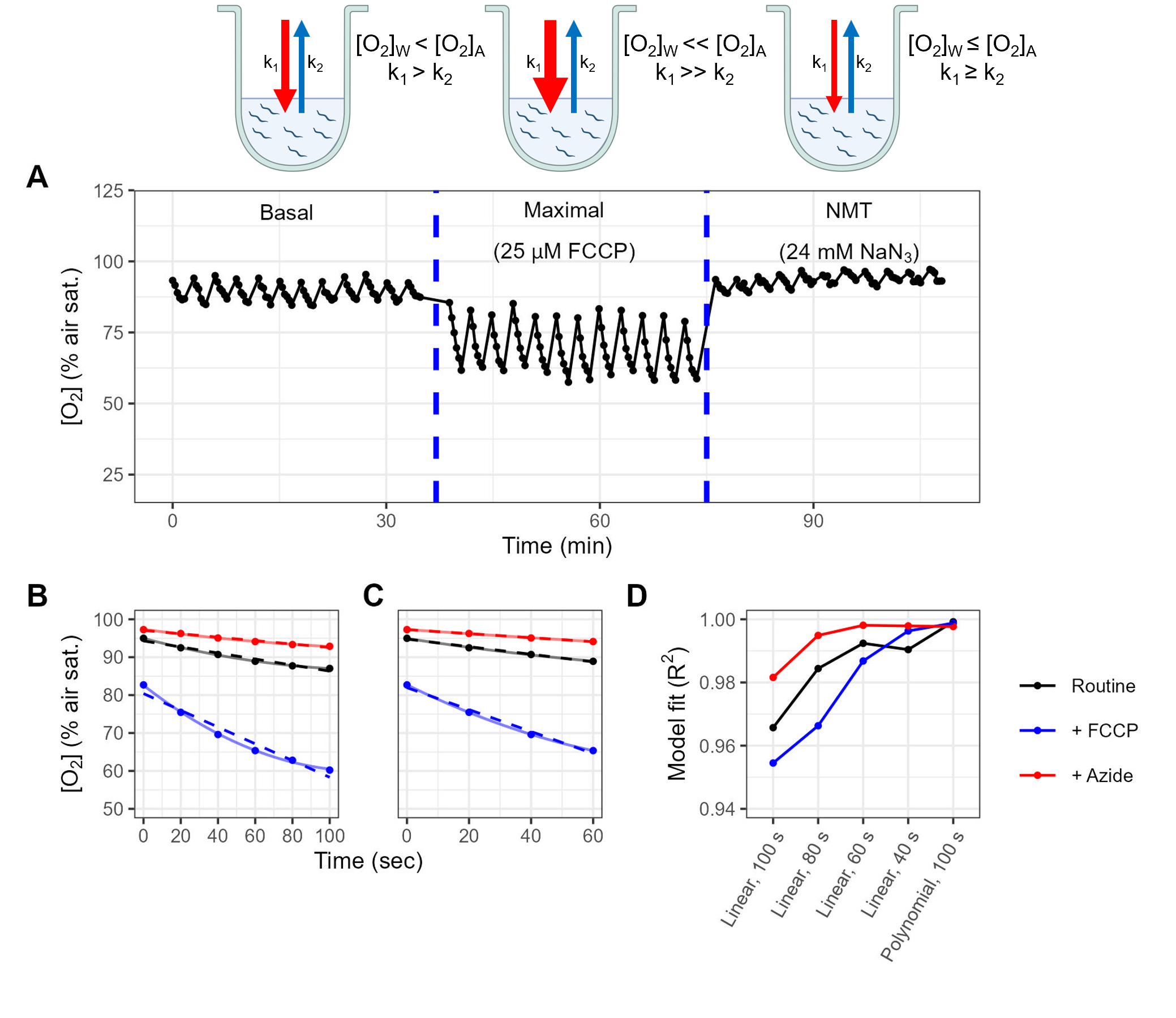
Figure S4. Determining the optimal window for the calculation of the *C. elegans* OCR. (A) Simplified models of oxygen diffusion under periods of moderate (basal), high (maximal) and low (non-mitochondrial, NMT) oxygen consumption in uncovered OxoPlate wells with an accompanying average oxygen trace of *C. elegans* samples treated sequentially (dashed lines) with FCCP and sodium azide (NaN_3_; N = 15 wells). During periods of low oxygen consumption, the oxygen concentration within each well ([O_2_]_w_) is similar to the oxygen concentration of the ambient air ([O_2_]_A_) resulting in similar rates of oxygen diffusion (k_1_ and k_2_) between the environment and the contents of each well. In contrast, during periods of high oxygen consumption, the [O_2_]_w_ drops significantly below the [O_2_]_A_, causing a large net influx of oxygen into the contents of each well, masking the biological OCR. These models are simplified to only consider the contents of each well and the ambient air. However, the material of the OxoPlates may act as an additional oxygen reservoir and may need to included in an accurate model of oxygen diffusion. (B-C) Oxygen concentrations averaged over each kinetic window displayed in panel A during basal, maximal and non-mitochondrial (NMT) respiration, with fitted curves of 2nd-order polynomials (solid lines) and linear regressions (dashed lines). Curves are fitted to the full measurement window in B, and the first 60 seconds in C. (D) Model fit (R^2^) of linear regressions using subsets of the full measurement window (100 s) in 20 s increments, compared to the model fit of a 2nd-order polynomial fit to the full measurement window.
