# Supplementary figures and images for: A novel C. elegans respirometry assay using low-cost optical oxygen sensors
Source: Biol Methods Protoc. 2025 Sep 30;10(1):bpaf072. doi: 10.1093/biomethods/bpaf072 (PMC12557035; doi:10.1093/biomethods/bpaf072)

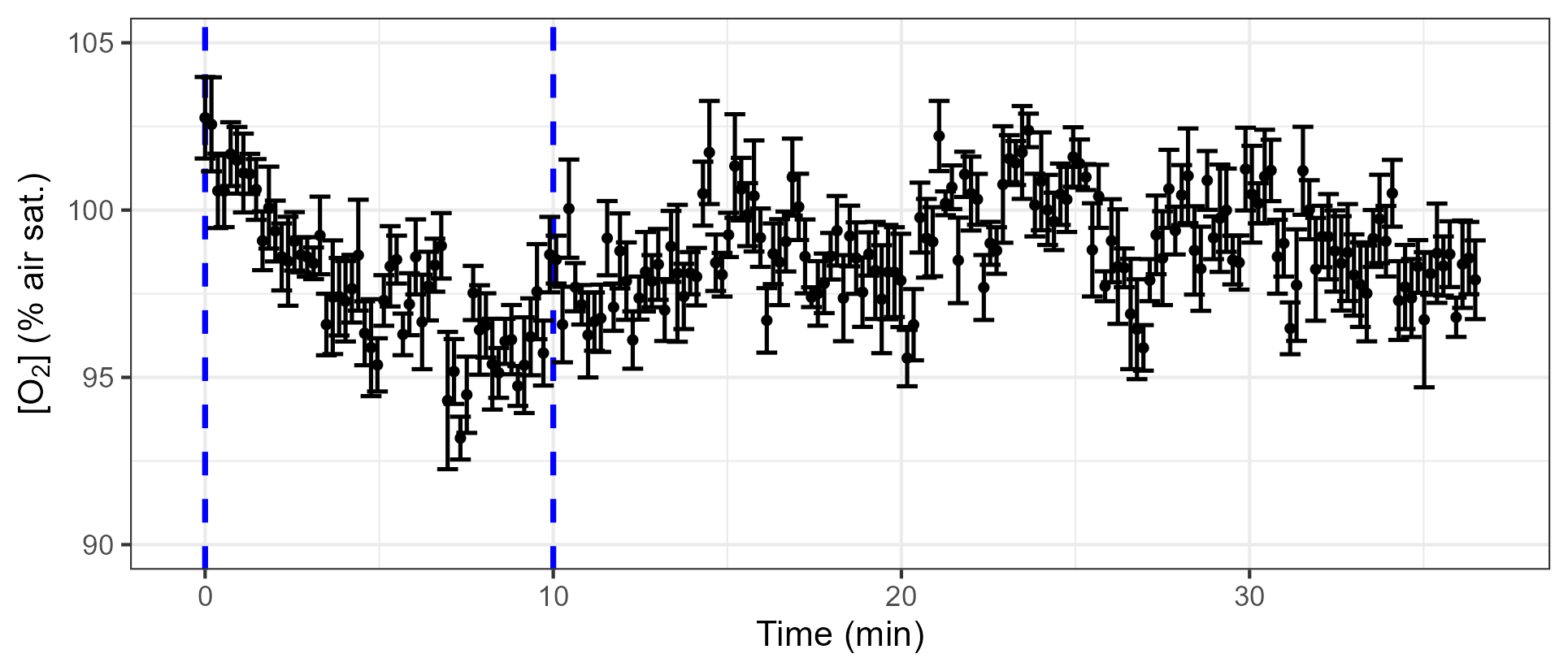

Supplement: bpaf072_Supplementary_Data [file bpaf072_supplementary_data.zip › Figure S1.tif]

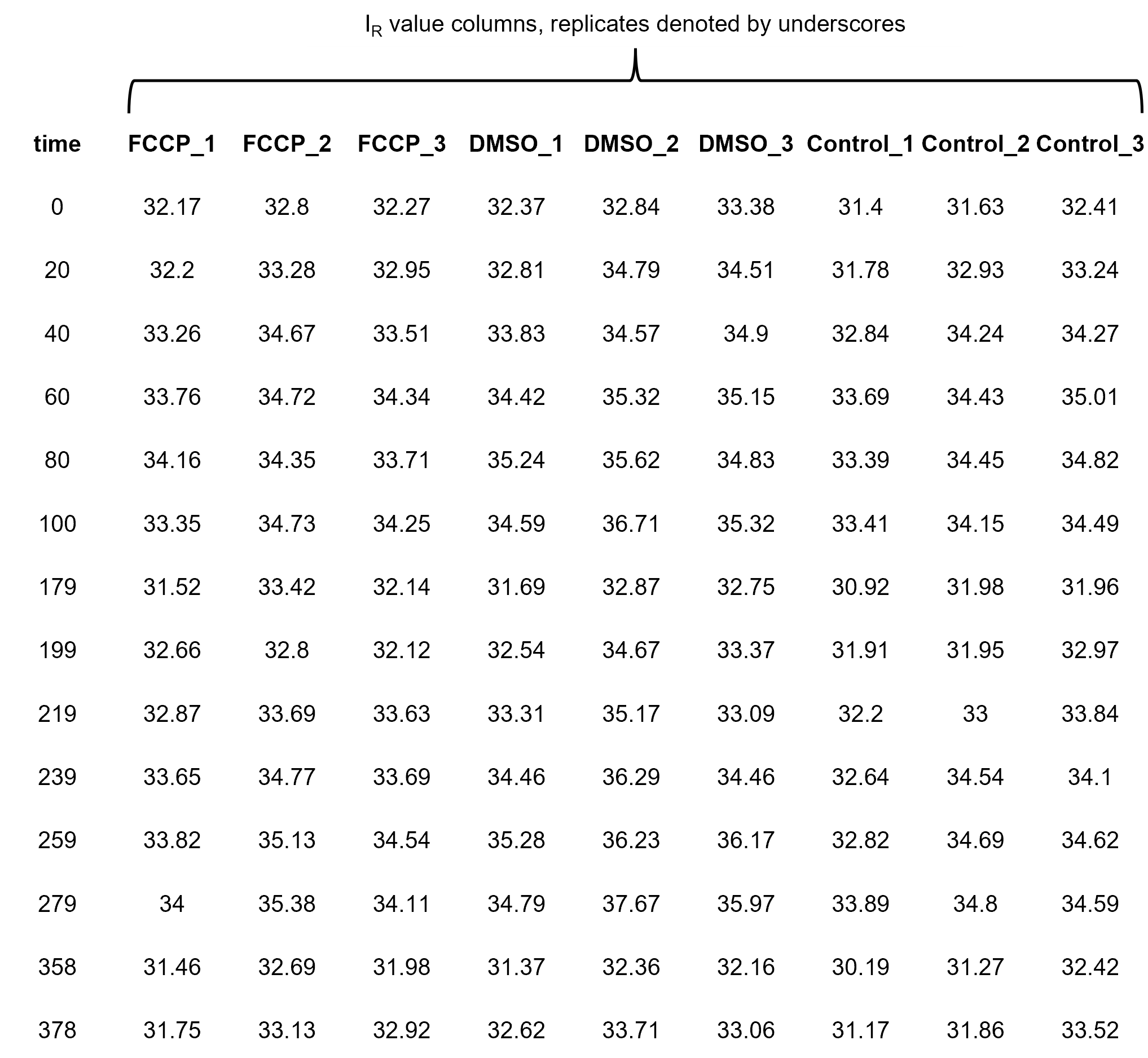

Supplement: bpaf072_Supplementary_Data [file bpaf072_supplementary_data.zip › Figure S2.tif]

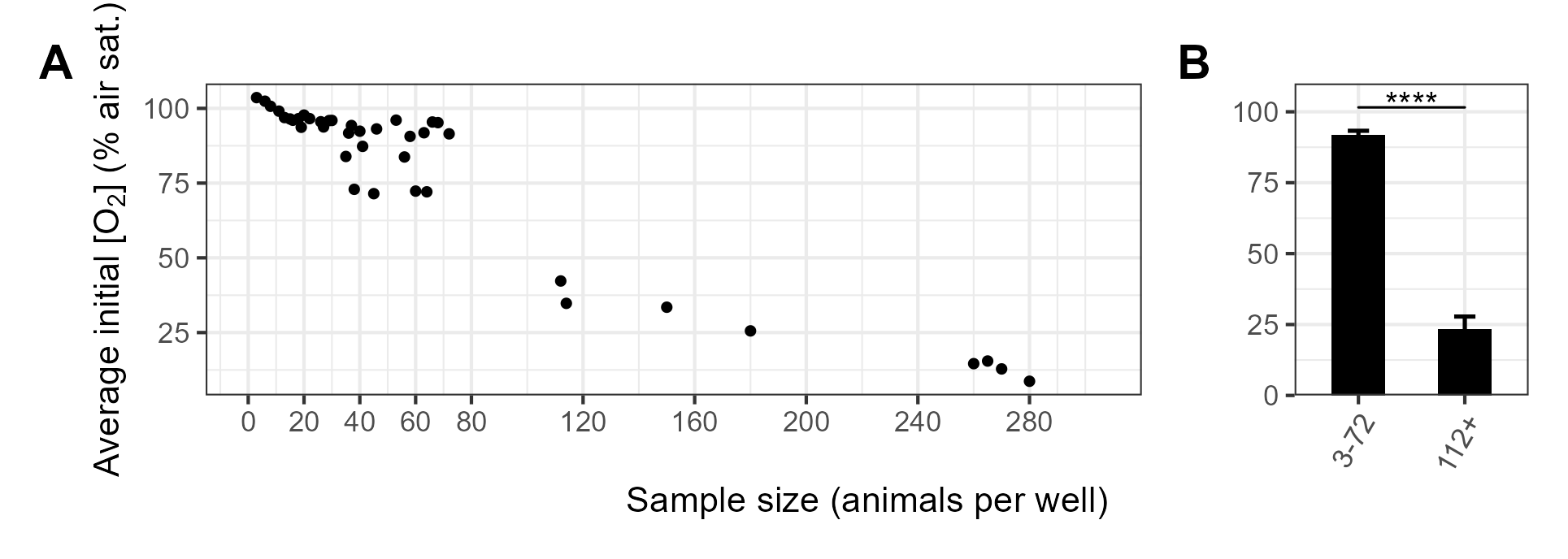

Supplement: bpaf072_Supplementary_Data [file bpaf072_supplementary_data.zip › Figure S3.tif]

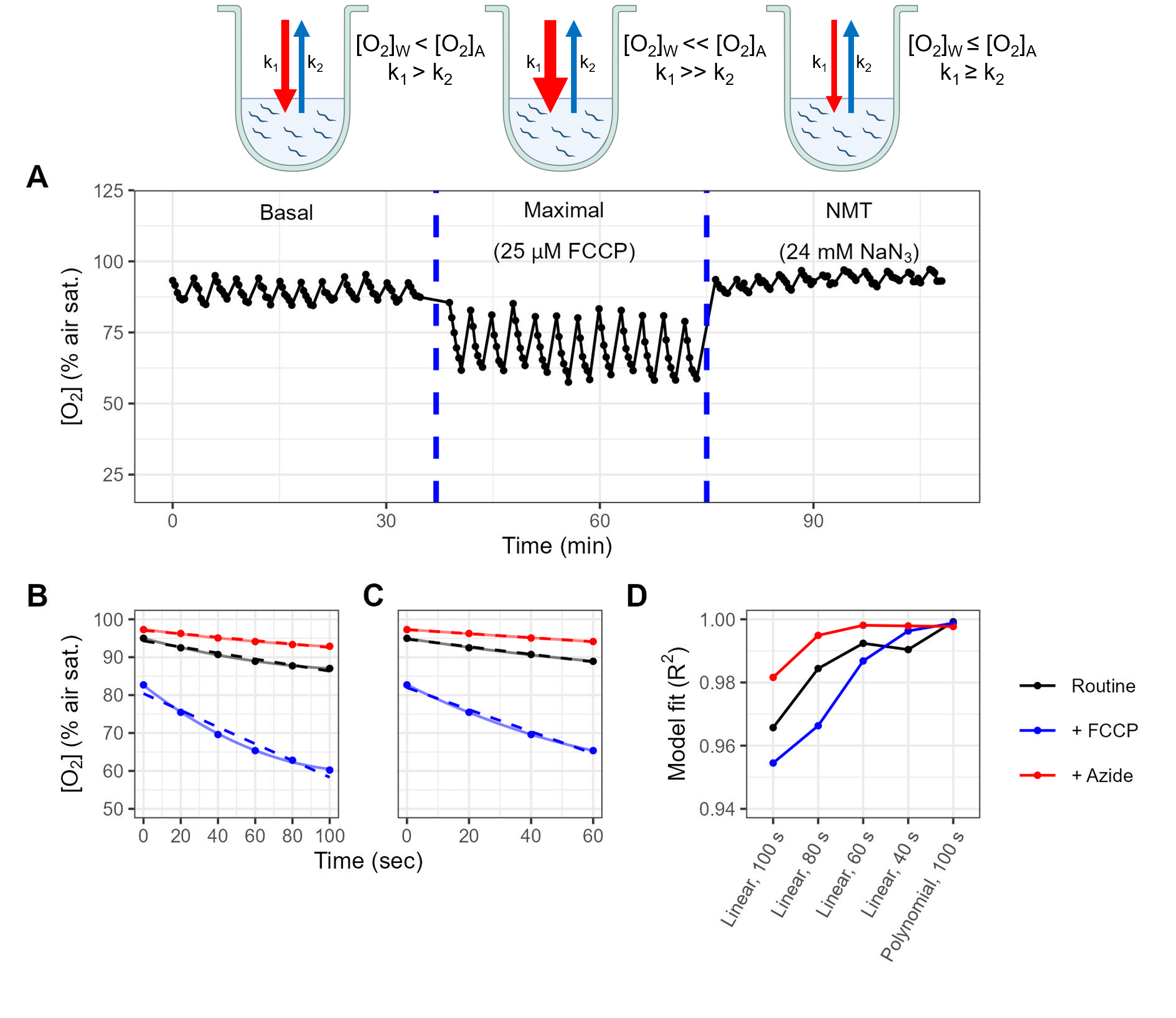

Supplement: bpaf072_Supplementary_Data [file bpaf072_supplementary_data.zip › Figure S4.tif]
